# Tracking Human Mobility Using WiFi Signals

**DOI:** 10.1371/journal.pone.0130824

**Published:** 2015-07-01

**Authors:** Piotr Sapiezynski, Arkadiusz Stopczynski, Radu Gatej, Sune Lehmann

**Affiliations:** 1 Department of Applied Mathematics and Computer Science, Technical University of Denmark, Kongens Lyngby, Denmark; 2 Media Lab, Massachusetts Institute of Technology, Cambridge, MA, United States of America; 3 Department of Economics, University of Copenhagen, Copenhagen, Denmark; 4 Niels Bohr Institute, University of Copenhagen, Copenhagen, Denmark; Beijing University of Posts and Telecommunications, CHINA

## Abstract

We study six months of human mobility data, including WiFi and GPS traces recorded with high temporal resolution, and find that time series of WiFi scans contain a strong latent location signal. In fact, due to inherent stability and low entropy of human mobility, it is possible to assign location to WiFi access points based on a very small number of GPS samples and then use these access points as location beacons. Using just one GPS observation per day per person allows us to estimate the location of, and subsequently use, WiFi access points to account for 80% of mobility across a population. These results reveal a great opportunity for using ubiquitous WiFi routers for high-resolution outdoor positioning, but also significant privacy implications of such side-channel location tracking.

## Introduction

Due to the ubiquity of mobile devices, the collection of large-scale, longitudinal data about human mobility is now commonplace [1]. High-resolution mobility of individuals and entire social systems can be captured through a multitude of sensors available on modern smartphones, including GPS and sensing of nearby WiFi APs (access points or routers) and cell towers. Similarly, mobility data may be collected from systems designed to enable communication and connectivity, such as mobile phone networks or WiFi systems (e.g. at airports or on company campuses) [2, 3]. Additionally, large companies such as Google, Apple, Microsoft, or Skyhook, combine WiFi access points with GPS data to improve positioning [4], a practice known as ‘wardriving’. While widely used, the exact utility and mechanics of wardriving are largely unknown, with only narrow and non-systematic studies reported in the literature [5, 6]. As a consequence, it is generally not known how WiFi networks can be used for sensing mobility on a societal scale; this knowledge is proprietary to large companies.

In the scientific realm, the mobility patterns of entire social systems are important for modeling spreading of epidemics on multiple scales: metropolitan networks [7–9] and global air traffic networks [10, 11]; traffic forecasting [12]; understanding fundamental laws governing our lives, such as regularity [13], stability [14], and predictability [15]. Predictability and stability of human mobility are also exploited by commercial applications such as intelligent assistants; for example Google Now [16] is a mobile application, which learns users’ habits to, among other services, conveniently provide directions to the next inferred location.

Mobility traces are highly unique and identify individuals with high accuracy [17]. Sensitive features can be extracted from mobility data, including home and work locations, visited places, or personality traits [18]. Moreover, location data are considered the most sensitive of all the commonly discussed personal data collected from or via mobile phones [19].

Here, we show that a time sequence of WiFi access points is effectively equal to location data. Specifically, having collected both GPS and WiFi data with high temporal resolution (median of 5 minutes for GPS and 16 seconds for WiFi) in a large study [20], we use six months of data for 63 participants to model how lowering the rate of location sampling influences our ability to infer mobility. The study participants are students with heterogeneous mobility patterns. They all attend lectures on campus located outside of the city center, but live in dormitories and apartments scattered across the metro area at various distances from the university.

By mapping the WiFi data, we are able to quantify details of WiFi-based location tracking, which are usually not available to the general public. We find that the geo-positioning inferred from WiFi access points (APs or routers) could boost efficacy in other data collection contexts, such as research studies. In addition, our findings have significant privacy implications, indicating that for practical purposes WiFi data should be considered location data. As we argue in the following sections, this finding is not recognized in current practices of data collection and handling.

## Methods

### The dataset

Out of the 130+ participants of the study [20], we selected 63 for which at least 50% of the expected data points are available. The methods of collection, anonymization, and storage of data were approved by the Danish Data Protection Agency, and complies both with local and EU regulations. Written informed consent was obtained via electronic means, where all invited participants read and digitally signed the form with their university credentials. The median period of WiFi scans for these users was 16 seconds, and the median period of GPS sampling was 10 minutes. The data spans a period of 200 days from October 1st, 2012 to April 27th, 2013.

### Known routers and coverage

In the article we use a simple model of locating the WiFi routers. We consider an access point as *known* if it occurred in a WiFi scan within one second of a GPS location estimation. The shortcomings of this approach and possible remedies are described in more detail in S1 File.

We define *time coverage* as a fraction of ten-minute bins containing WiFi data in which at least one *known router* was scanned. For example, let us assume that the user has data in 100 out of 144 timebins during a day, and in 80 of these timebins there is a known router visible. Therefore, that user’s coverage for that day is 80%. The average time coverage for a day is the mean coverage of all users who had any WiFi information in that day. This way our results are independent from missing data caused by imperfections in data collection system deployed in the study.

**Fig 1:**
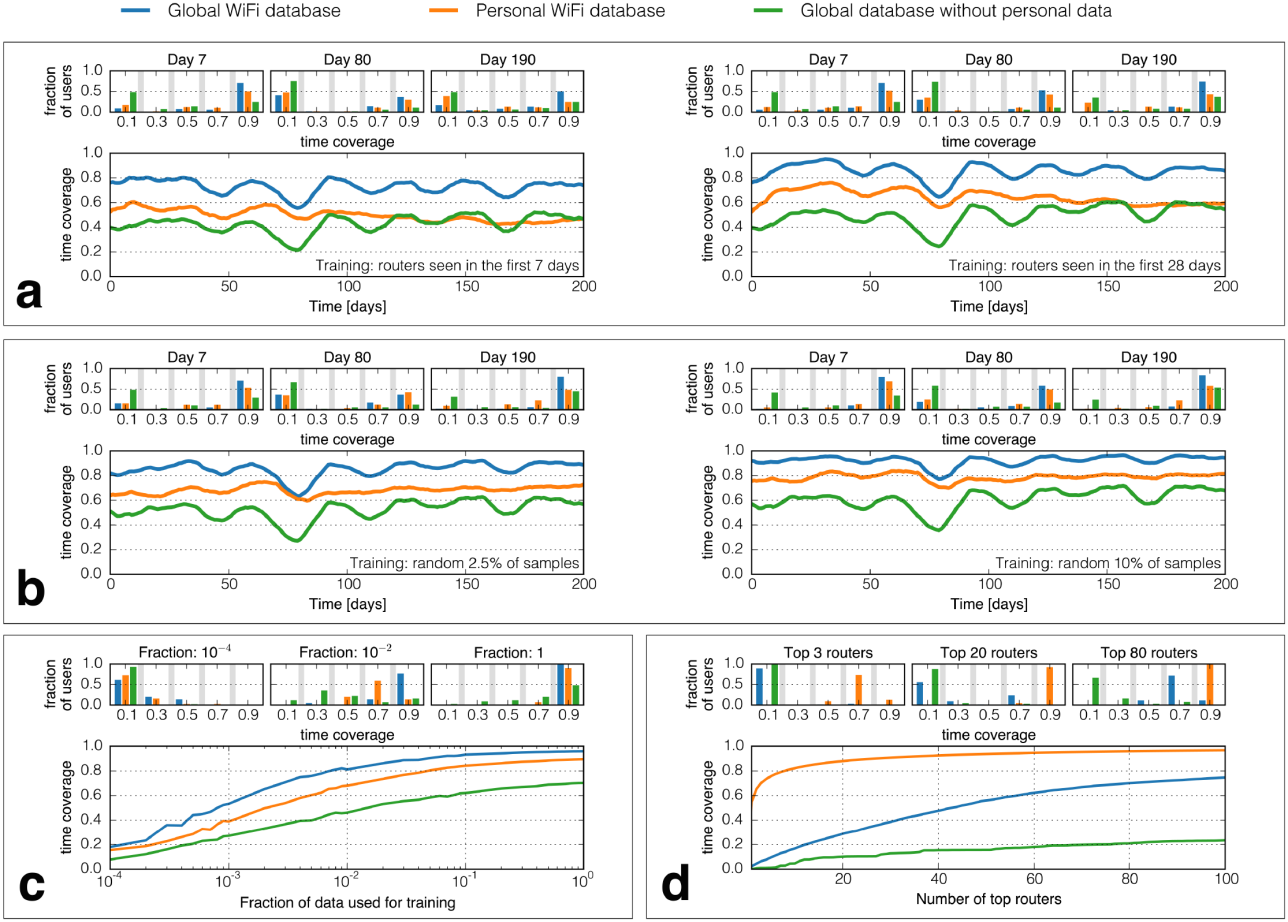
The time coverage provided by the routers with known position depends on who collects the corresponding location data and when it happens. In each subplot the orange line describes the scenario where each individual collects data about themselves and does not share it with others; the blue line corresponds to a system in which the location of routers discovered by one person is made known to other users; the green line presents a situation where each individual can use the common pool of known routers but does not discover access points herself. **a. Stability of location**. Learning the location of APs seen during the first seven (left panel) or 28 (right panel) days, leads to performance gradually decreasing with time in the personal case (orange line). The histograms of time coverage distribution for day 190 show that this decline is driven by a growing number of people who spend only ∼10% of time in the locations they visited in the beginning of the observation. The global approach (blue line) does not show this tendency, which indicates that people rotate between common locations rather than moving to entirely new places. **b, c. Representativeness of randomly selected locations.** Random subsampling with an average period of 24 hours (less than 1% of all available location estimations) is sufficient to find the most important locations in which people spend more than 80% of their time; using an average period of 4 hours (2.5% percent of all available location data) results in ∼85% coverage. The personal database does not expire since the location is sampled throughout the experiment, not only in the beginning. **d. Limited number of important locations.** Although querying commercial services for WiFi geolocation is costly, knowing the location of only the 20 most prevalent routers per person in the dataset results in an average coverage of ∼90%. Since people’s mobility overlaps, there is a benefit of using a global database rather than treating all mobility disjointly.

In Fig 1 we present three different approaches to sampling, which we describe here in detail. **Initial-period sampling.** As presented in Fig 1a, we learn the location of the routers sequentially. With each GPS location estimation accompanied with a WiFi scan, we add the visible access points to the list of known routers. The learning curve can be observed for the first seven days (Fig 1a, left panel) or the first 28 days (Fig 1a, right panel). **Random subsampling.** In the random subsampling scenario we select a set fraction of available GPS location estimations, each paired with a WiFi scan. Each GPS estimation provides information on the position of all routers seen in the paired scan. This scenario can be realized after the data collection is finished, as the location estimations are used to locate the WiFi scans which happened both before and after said estimations. The results are presented in Fig 1b. **Top routers.** We select the top routers in a greedy fashion after the data collection is finished. We sort the routers in descending order by the number of user timebins they occur in. We choose the top one router, and then we select the routers which provide the biggest increase in the number of user timbebins covered. Due to high density of access points, each semantic place is described by presence of several routers, but location of only one of them has to be established to find the geographic position of the place. In this sampling method we do not rely on our own GPS data—top routers are found purely based on their occurrence in the WiFi scans, regardless of availability of GPS scans within the one second time delta. The results of such sampling are presented in Fig 1e.

### Data collection scenarios

Each subplot in Fig 1 contains series coming from three different simulated collection scenarios. In the **global** scenario, there is a pool of WiFi routers locations estimations coming from all users, and a router is considered known if at least one person has found its location. This scenario simulates the function of such services as for example mobile Google Maps. In the **personal** scenario each user can only use their own data, a router can be known to them only if they found its location themselves. It simulates collecting data in a disjoint society, where each person frequents different locations. Finally, in the **global with no personal data** scenario, each user can exploit estimations created by everybody else, but without contributing their own data.

## Results

Ubiquitously available WiFi access points can be used as location beacons, identifying locations based on BSSID (basic service set identifier, uniquely identifying every router) broadcast by APs. These locations are not intrinsically geographical, as the APs do not have geographical coordinates attached. However, since the placement of APs tends to remain fixed, mapping an AP to a location where it was seen once is sufficient to associate all the subsequent scans from the user device with geographical coordinates. See S1 File for details on inferring the geographical locations of routers, as well as identifying (and discarding data from) mobile access points.

**Fig 2:**
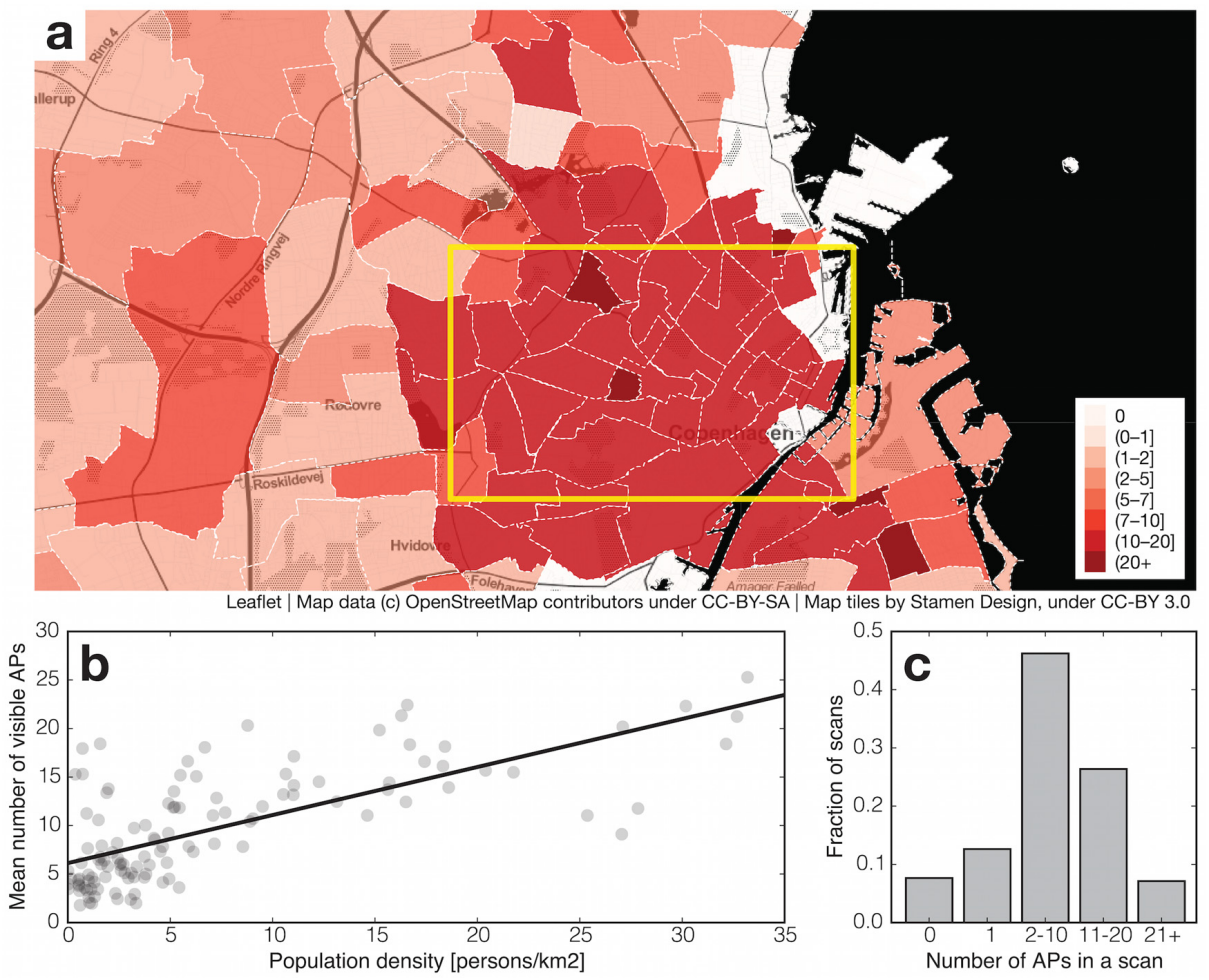
WiFi routers are located where people live. a: Map of Greater Copenhagen Area, divided into parishes with color indicating average number of routers discovered per scan; rectangle overlay indicates the city center. b: The number of access points visible in each scan is correlated with the population density (*r* ^2^ = 0.5). Even in low population density areas there are several routers visible on average in each scan. Therefore, knowing the positions of only a subset of routers is enough for precise location sensing. c: Distribution of number of routers per scan. In our dataset 92% of scans contain at least one router.

WiFi networks are ubiquitous. In our population, 92% of all WiFi scans detect at least one access point, and 33% detect more than 10 APs, as shown in Fig 2c. In densely-populated areas, an average of 25 APs are visible in every scan, with population density explaining 50% of the variance of the number of APs, as shown in Fig 2b. WiFi scans containing at least one visible AP can be used for discovering the location of the user, with a typical spatial resolution on the order of tens of meters.

We investigate three approaches to using access points as location beacons, all of which enable WiFi-based location tracking even with limited resources: (1) recovering APs’ locations from mobility traces collected during an initial training period (exploiting the long-term stability of human mobility), (2) recovering APs’ locations from randomly sampled GPS updates (exploiting low entropy of human mobility, see S1 File for distinction between stability and low entropy), and (3) using only the most frequently observed APs for which location can be feasibly obtained from external databases. The task is to efficiently assign geographical coordinates (latitude and longitude) to particular APs, so they can be used as beacons for tracking user’s location. In the following sections, we refer to *time coverage* as the fraction of ten-minute timebins, in which at least one router with a known location is observed.

### Stability of human mobility allows for efficient WiFi-based positioning

Human mobility has been shown to remain stable over long periods of time [13]. We find that participants in our study have stable routines, with locations visited in the first one, two, three, and four weeks of the study still visited frequently six months later. Learning the locations of routers seen during the first seven days (corresponding to ∼3.5% of the observations, shown in Fig 1a, left panel) provides APs’ locations throughout the rest of the experiment sufficient for recovering ∼55% of users mobility until the Christmas break around days 75–90. When the location of routers seen by each person is inferred using only this person’s data (the personal-only WiFi database case, shown using an orange line in Fig 1), the information expires with time: there is a stable decrease in time coverage after Christmas break. This decline is evident both when a week (Fig 1a, left panel) and four weeks (right panel) are used for training, with the time coverage dropping ∼18 percentage points between days 60 and 160. The histograms above each plot show the distribution of time coverage in selected points in time (at 7, 80, 190 days respectively). The distribution for day 190 reveals that the expiry of the personal database validity is driven by individuals who significantly altered routines, with 40% of participants spending only around 10% of time in locations they have visited in the first week. In contrast, when the inferred locations of routers are shared among people (the global database case, represented by a blue line) the information does not expire and shows no decreasing trend during the observation period. This implies that rather than moving to entirely new locations, people begin to visit places that are new to them, but familiar to other participants. The histograms of time coverage distribution in both panels of Fig 1a reveal that the individuals are heterogeneous in their mobility. The coverage in most cases is highly affected in the non-personal case (where the person does not collect their own location information, but data from others is used, marked using green in the figures), but 20% of participants retain a coverage of above 80% throughout the observation period, see Fig 1a, left panel. People living and working close to each other (like students in a dormitory) share a major part of their mobility and thus location of the APs they encounter can be estimated using data collected by others.

The demonstrated stability of human mobility patterns over long periods has real-life privacy implications. Denying a mobile application access to location data, even after a short period, may not be enough to prevent it from tracking user’s mobility, as long as its access to WiFi scans is retained.

### Human mobility can be efficiently captured using infrequent location updates

Sampling location randomly across time (Fig 1b), rather than through the initial period (Fig 1a) provides a higher time coverage, which is retained throughout the observation. With around one sample per day per person on average, the location can be inferred 80% of the time in case of global lookup base and 70% in personal case (see Fig 1c, at training fraction of 0.007).

The histograms in Fig 1b confirm that distribution of coverage in the non-personal case is bimodal within our population: mobility of some individuals can effectively be modeled using data from people around them, while patterns of others are so distinct they require using self-collected information. The single-mode distribution of coverage in the personal case and the fact that the distribution is unchanged between day 7 and day 190 show the lack of temporal decline when sampling happens throughout the observation period.

The GPS sensor on a mobile device constitutes a major battery drain when active [21], whereas the WiFi frequently scans for networks by default. Our results show that GPS-based location sampling rate can be significantly reduced in order to save battery, while retaining high resolution location information through WiFi scanning. Our analyses also point to another scenario where WiFi time series can result in leaks of personal information. Infrequent location data can be obtained from a person’s (often public) tweets, Facebook updates, or other social networking check-ins and then matched with their WiFi records to track their mobility.

### Overall human mobility can be effectively captured by top WiFi access points

As previously suggested [15], people’s mobility has low entropy and thus a few most prevalent routers can work effectively as proxies for their location. Fig 1d shows that inferring the location of just 20 top routers per person on average (which, given the median count of 22 000 routers observed per person, corresponds to 0.1% of all routers seen) translates to knowing the location of individuals 90% of the time. Since our population consists of students, who attend classes in different lecture halls in various buildings across the campus, we expect that the number of access points necessary to describe mobility of persons with a fixed work location can be even lower. There are persons in our study, for whom just four access points correspond to 90% of time coverage (see Fig D in S1 File for details).

That the mobility of individuals in our sample overlaps is apparent in Fig 1d as the time coverage of three top routers in the personal case is the same as in the global coverage using the total of 80 routers (instead of 189 disjoint routers).

As a consequence, a third party with access to records of WiFi scans and no access to location data, can effectively determine the location of each individual 90% of time by sending less than 20 queries to commercial services such as Google Geolocation API or Skyhook.

### Single-user analysis

**Fig 3:**
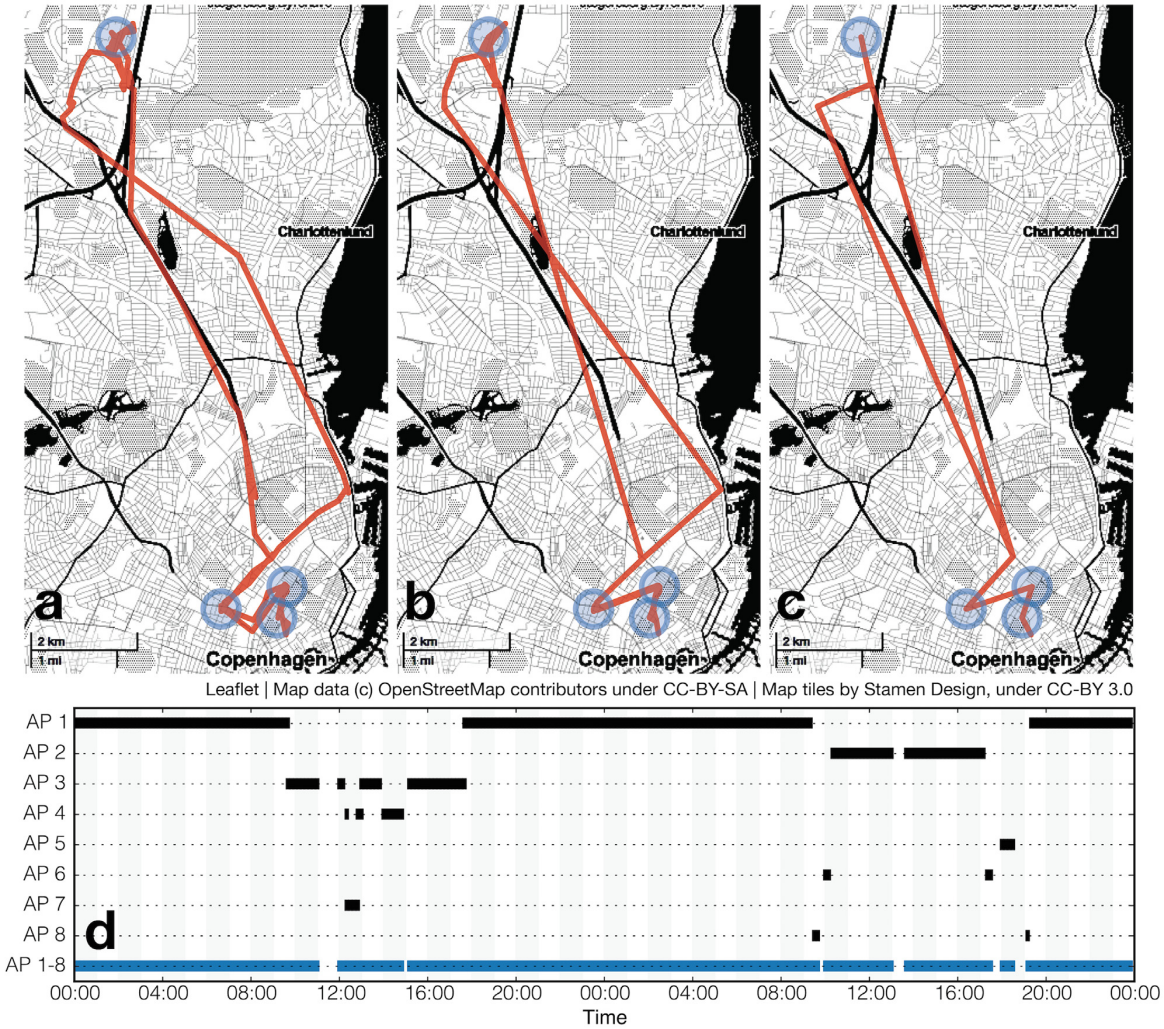
48 hours of location data of one of the authors, with the four visited locations visited marked in blue: home, two offices, and a food market. Even though the author’s phone has sensed 3 822 unique routers in this period, only a few are enough to describe the location more than 90% of time. a. traces recorded with GPS; b. traces reconstructed using all available data on WiFi routers locations—the transition traces are distorted, but all stop locations are visible and the location is known 97% of the time. c. with 8 top routers it is still possible to discover stop locations in which the author spent 95% of the time. In this scenario transitions are lost. d. timeseries showing when during 48 hours each of the top routers were seen. It can be assumed that AP 1 is home, as it’s seen every night, while AP 2 and AP 3 are offices, as they are seen during working hours. The last row shows the combined 95% of time coverage provided by the top 8 routers.

To illustrate the ubiquity of WiFi access points and how effectively they can be used to infer mobility patterns, we present a small example dataset containing measured and inferred location information of one of the authors, collected over two days. During the 48 hours of observation, the researcher’s phone was scanning for WiFi with a median period of 44 seconds, measuring on average 19.8 unique devices per scan, recording 3 822 unique access points. Only one scan during the 48 hours was empty, and one scan yielded 113 unique results. Fig 3a shows the corresponding GPS trace collected with a median sampling period of 5 minutes. When dividing the 48 hours of the test period into 10 minute bins, a raw GPS trace provides location estimation in 89% of these bins. Four stop locations are marked with blue circles and include home, two offices, and a food market visited by the researcher. Fig 3b shows the estimation of this trace based on the inferred locations of WiFi routers, see S1 File for detailed information on the location inference. The four stop locations are clearly visible, but the transitions have lower temporal resolution and errors in location estimations. This method provides location information in 97% of temporal bins. Using WiFi increases overall coverage, but might introduce errors in location estimation of routers which were only observed shortly, for example during transition periods. Fig 3c shows the estimation of this trace based on the locations of top 8 (0.2%) WiFi routers. The four important locations have been correctly identified, but information on transitions is lost. Information in 95% of temporal bins is available. Finally, Fig 3d shows a graphical representation of how much time the researcher spends in any one of the top eight locations during the observation time. Note that the first four locations account for an overwhelming fraction of the 48 hours.

Knowing the physical position of the top routers and having access to WiFi information reveals the location of the user for the majority of the timebins. The details of trajectories become lost as we decrease the number of routers we use to estimate locations. With too few routers might not be possible to determine which of possible routes the subject chose or how long she took to travel through each segment of the trip. On the other hand, the high temporal resolution of the scans allows for very precise discovery of arrival and departure times and of time spent in transit. Such information has important implications for security and privacy, as it can be used to discover night-watch schedules, find times when the occupants are not home, or efficiently check work time of the employees.

## Discussion

Our world is becoming progressively more enclosed in infrastructures supporting communication, mobility, payments, or advertising. Logs from mobile phone networks have originally been considered only for billing purposes and internal network optimization; today they constitute a global database of human mobility and communication networks [13]. Credit card records form high-resolution traces of our spending behaviors [22]. The omnipresent WiFi networks, intended primarily for communication, has now became a location tracking infrastructure, as described here. The pattern is clear: every new cell tower, merchant with credit card terminal, every new private or municipal WiFi network offer benefits to the connected society, but, at the same time, create opportunities and perils of unexpected tracking. Cities entirely covered by WiFi signal provide unprecedented connectivity to citizens and visitors alike; at the same time multiple parties have to incorporate this fact in their policies to limit privacy abuse of such infrastructure. Understanding and quantifying the dynamics of privacy and utility of infrastructures is crucial for building connected and free society.

Since the creation of comprehensive databases containing geolocation for APs is primarily carried out by large companies [4], one might assume that WiFi based location tracking by ‘small players’, such as research studies or mobile applications, is not feasible. As we have shown above, however, APs can be very efficiently geolocated in a way that covers a large majority of individuals’ mobility patterns.

In the results, we focused on outdoor positioning with spatial resolution corresponding to WiFi AP coverage: we assume that if at least one AP is discovered in a scan, we can assign the location of this AP to the user. This is a deliberately simple model, described in detail in S1 File, but we consider the resulting spatial resolution sufficient for many aspects of research, such as studying human mobility patterns. The spatial resolution of dozens of meters is higher than for example CDR data [13], which describes the location with the accuracy of hundreds of meters to a few kilometers. Incorporating WiFi routers as location beacons can aid research by drastically increasing temporal resolution without additional cost in battery drain.

Students live in multiple dormitories on and outside of campus, take multiple routes commuting to the university, frequent different places in the city, travel across the country and beyond. While the students spend most of their time within a few dozens of kilometers from their homes, they also make international and intercontinental trips (see Figs B and C in S1 File for details). Such long distance trips are not normally captured in studies based on telecom operator data. Our population is densely-connected and in this respect it is biased, in the same sense as any population of people working in the same location. We do simulate a scenario in which the individuals do not form a connected group by analyzing the results for personal-only database. We expect the obtained results to generalize outside of our study.

Our findings connect to an ongoing debate about the privacy of personal data [23]. Location data has been shown to be among the most sensitive categories of personal information [19]. Still, a record of WiFi scans is, in most contexts, not considered a location channel. In the Android ecosystem, which constitutes 85% of global smartphone market in Q2 2014 [24], the permission for applications to passively collect the results of WiFi scans is separate from the location permission; moreover, the *Wi-Fi connection information (ACCESS_WIFI_STATE)* permission is not considered ‘dangerous’ in the Android framework, whereas both high-accuracy and coarse location permissions are tagged as such [25]. While it has been pointed out that Android WiFi permissions may allow for inference of sensitive personal information [26], the effect has not been quantified through real-world data. Here we have shown that inferring location with high temporal resolution can be efficiently achieved using only a small percentage of the WiFi APs seen by a device. This makes it possible for any application to collect scanned access points, report them back, and inexpensively convert these access points into users’ locations. The impact is amplified by the fact that apps may passively obtain results of scans routinely performed by Android system every 15–60 seconds. Such routine scans are even run when the user disables WiFi. See S1 File for additional analysis on data privacy in the Android ecosystem.

Developers whose applications declare both location and WiFi permissions are able to use WiFi information to boost the temporal resolution of any collected location information. We have shown that even if the location permission is revoked by the user, or removed by the app developers, an initial collection of both GPS and WiFi data is sufficient to continue high-resolution tracking of the user mobility for subsequent months. Many top applications in the Play Store request *Wi-Fi connection information* but not explicit location permission. Examples from the top charts include prominent apps with more than 100 million users each, such as Candy Crush Saga, Pandora, and Angry Birds, among others. We are not suggesting that these or other applications collect WiFi data for location tracking. These apps, however, do have a *de facto* capability to track location, effectively circumventing Android permission model and general public understanding.

Due to uniqueness of location traces, users can be easily identified across multiple datasets [17]. Our results indicate that any application can use WiFi permission to link users to other public and private identities, using data from Twitter or Facebook (based on geo-tagged tweets and posts), CDR data, geo-tagged payment transactions; in fact any geo-tagged data set. Such cross-linking is another argument why WiFi scans should be considered a highly sensitive type of data.

In our dataset, 92% of WiFi scans have at least one visible AP. Even in the most challenging scenario, when there are no globally shared locations and each individual frequents different places, top 20 WiFi access points per person can be efficiently converted into geolocations (using Google APIs or crowd-sourced data) and used as a stable location channel. These results should inform future thinking regarding the collection, use, and data security of WiFi scans. We recommend that WiFi records be treated as strictly as location data.

## Supporting Information

S1 File Additional details on the properties of the data and the employed analysis methods. In this Supporting File we present an example method of inferring the locations of WiFi routers, explain the interplay between the long term stability and low entropy of human mobility, provide a detailed description of the mobility properties of the participants (**Figs B** and **C**), show the distributions of time coverage of top routers (**Fig D**), and explain how Android permission model allows apps to access the WiFi information of the user.
(PDF)
