# Supplementary material for: Tracking Human Mobility Using WiFi Signals
Source: PLoS One. 2015 Jul 1;10(7):e0130824. doi: 10.1371/journal.pone.0130824 (PMC4489206; doi:10.1371/journal.pone.0130824)
Supplement: S1 File — In this Supporting File we present an example method of inferring the locations of WiFi routers, explain the interplay between the long term stability and low entropy of human mobility, provide a detailed description of the mobility properties of the participants (Figs B and C), show the distributions of time coverage of top routers (Fig D), and explain how Android permission model allows apps to access the WiFi information of the user. (PDF) [file pone.0130824.s001.pdf]

Tracking Human Mobility using WiFi signals

Supplementary Information

Piotr Sapiezynski Arkadiusz Stopczynski Radu Gatej Sune Lehmann

Inferring location of routers.

In the article we use a deliberately simplistic model of locating the WiFi routers. We assume that if we find a WiFi scan and a GPS location estimation which happened within a one second time difference we can assume that all routers visible in the scan are at the geographical location indicated by the GPS reading. Due to effective outdoor range of WiFi routers of approximately 100 meters, this assumption introduces an obvious limitation of accuracy of location inference. Moreover, there are a number of mobile access points such as routers installed in public transportation or smartphones with hotspot capabilities. Such devices cannot effectively be used as location beacons and will introduce noise into location estimations unless identified and discarded. We propose and test the following method. For each GPS location estimation with timestamp t_{GPS} we find WiFi scans performed by the same device at t_{WiFi} so that $t_{GPS} - 1s \leq t_{WiFi} \leq t_{GPS} + 1s$ and select the one, for which $|t_{GPS} - t_{WiFi}|$ is the smallest. We then add the location estimation and its timestamp to the list of locations where each of the available WiFi access points was seen. For each device, we fit a density-based spatial clustering of applications with noise (DBSCAN) model [1] specifying 100 meters as the maximum distance parameter ε. If there are no clusters found, or the found clusters contain less than 95% of all locations associated with the said router we assume the router is mobile and to be discarded from further analysis. If only one cluster is detected and it contains at least 95% of all points, we assume the geometric median of these points is the physical location of the router. If there are more clusters found and they contain at least 95% of all points, we verify if these clusters are disjoint in time: if the timestamps of sightings do not overlap between those clusters, we assume the device is a static access point which has been moved to a different place during the experiment. Otherwise, we classify the access point as a mobile device and do not use it as a location proxy.

In the proposed method we assume accuracy of tens of meters is satisfactory, and hence do not find a need to exploit the received signal strength information [2]. Arguably, with the sparse data that we operate on, employing received signal strength could lead to more confusion, as it can vary greatly for one location, depending on the position of the measuring smartphone, and presence of humans and other objects obstructing the signal. Fig A shows timeseries of signal strengths received by a non-moving smartphone, which vary as much as 10 dB, which corresponds to drastic differences in estimated distance to the source, as in free-space propagation model extending the distance $\sqrt{10} \approx 3.16$ times corresponds to 10 dB loss in received signal strength.

In the 200 days of observations, the participants have scanned 487 216 unique routers, out of which 64 983 were scanned within a second of a GPS estimation. As many as 57 912 were only seen less than five times which we assumed to be the minimum number of sightings to be considered a cluster, which left only 7 071 routers for further investigation. In 1 760 cases there were no clusters found, or there was more than 5% noise. In 5 267 cases there was only one cluster and less than 5% of noise. Out of 21 cases there were multiple clusters and less than 5% of noise, 9 revealed no time overlap between clusters. We verified our heuristic of determining which routers are mobile by classifying routers which are very likely mobile, as their networks are called AndroidAP (default SSID for a hotspot on Android smarphones), iPhone (default SSID for iPhones), Bedrebustur or Commutenet (names of networks on buses and trains in Copenhagen). Out of 340 such devices 323, or 95%, were identified as mobile, and 17 as fixed-location devices.

All in all, out of 487 216 unique APs we believe we managed to estimate the location of 5 276, we identified 1 771 as mobile, and did not have enough data to investigate 480 169. Even though we only know the location of approximately 1% of all sensed routers, this knowledge is enough to estimate the location of users in 87% ten-minute timebins in the dataset.

Long term stability and low entropy of human mobility.

Long-term stability in the context of human mobility means that individuals keep returning to the same locations over long time periods. Arguably, most people do not often move, change the work place, or find

an entirely new set friends to visit. We use entropy in Shanon's definition, as presented in equation (1)

$$H(X) = -\sum_{i} P(x_i) \log P(x_i), \tag{1}$$

where X is the set of all possible locations, and $P(x_i)$ is the probability of a person being at location i. Therefore, the bigger the fraction of time a person spends in their top few places, the lower the entropy value of that person's mobility. In this sense, long-term stability is necessary for the low entropy, and both contribute to the predictability of human mobility.

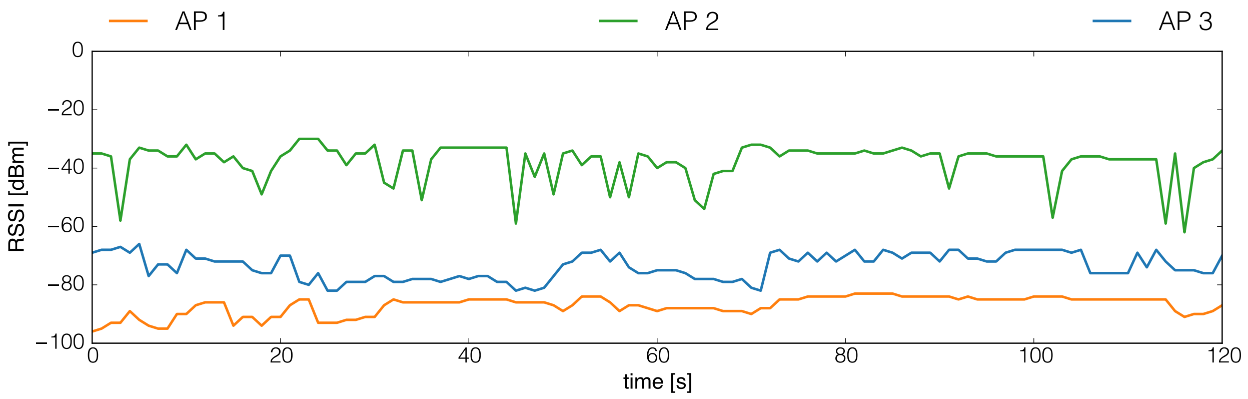

Fig A: Received signal strength can vary greatly even if the smartphone and the access points do not move.

Mobility of the studied population.

This article focuses on a population of students at a university. To show that their mobility is not constrained to the campus only, we present summary statistics about their mobility. Displacements in our dataset can be as big as 10 000 km. Given such extreme statistics, the radius of gyration, while commonly used in literature to describe mobility on smaller scales [3], is not a suitable measure here. Instead, in Fig B we show a qualitative overview in form of a heatmap of observed locations, as well as a distribution of time spent as a function of distance from home. For simplicity, we define the home location for each student as the location of the most prevalent access point in their data. We then calculate the median distance from home for each hour of the observation using their location data. For a more detailed view, we present the distribution for 48 randomly chosen students in Fig C.

Time coverage of top routers.

In this section we present a more detailed view on time coverage of top routers selected separately for each person. Fig DA shows the fraction of time which participants spent near to one of their top 20 routers. It is worth noting, that while home location is immediately apparent, there seems to be no definite "work" location in our population. This can be attributed to the fact that the participants of the observation are students who attend classes in different buildings and lecture halls and do not have an equivalent of an office. Fig DB is an enriched version of Fig 2d from the main text of the article. It shows that even though 20 routers are needed on average to capture 90% of mobility, there are participants for whom just four routers suffice.

Android Permissions.

The scope of Android permission *ACCESS_WIFI_STATE* is described in the developer documentation as "allows applications to access information about Wi-Fi networks" [4]. This permission provides the requesting application with a list of all visible access points along with their MAC identifiers after each scan ordered by any application on the phone (via broadcast mechanism). Moreover, with this permission the applications can start in the background when the first WiFi scan results appear after the phone boots: the app's BroadcastReceiver is called and the data can be collected without explicit *RECEIVE_BOOT_COMPLETED* permission. Requesting a WiFi scan requires the *CHANGE_WIFI_STATE* permission, marked as dangerous, but in most cases it is not necessary to request it: the Android OS by default performs WiFi scans in the intervals of tens of seconds, even when the WiFi is turned off; the setting to *disable* background scanning when WiFi is off is buried in the advanced settings.

Application developers often use *ACCESS_WIFI_STATE* to obtain information whether the device is connected to the Internet via mobile or WiFi network. This information is useful, for example, to perform larger downloads only when the user in connected to a WiFi network and thus avoid using mobile data. This is an unnecessarily broad permission to use for this purpose, as the same information can be obtained

with *ACCESS_NETWORK_STATE*, which provides all the necessary information without giving access to personal data of WiFi scans:

```
ConnectivityManager cManager =
        (ConnectivityManager) getSystemService(Context.CONNECTIVITY_SERVICE);
NetworkInfo mWifi = cManager.getNetworkInfo(ConnectivityManager.TYPE_WIFI);
if (mWifi.isConnected()) { } //wifi is connected
```

Since the *ACCESS_WIFI_STATE* together with *INTERNET* permission (for uploading the results) are effectively sufficient for high-resolution location tracking, we suggest the developers transition to using the correct permissions and APIs for determining connectivity and that accessing the result of WiFi scan requires at least the *ACCESS_COARSE_LOCATION* permission.

References

[1] Ester M, peter Kriegel H, S J, Xu X. A density-based algorithm for discovering clusters in large spatial databases with noise. AAAI Press; 1996. p. 226–231.

[2] Liu H, Darabi H, Banerjee P, Liu J. Survey of wireless indoor positioning techniques and systems. Systems, Man, and Cybernetics, Part C: Applications and Reviews, IEEE Transactions on. 2007;37(6):1067–1080.

[3] Gonzalez MC, Hidalgo CA, Barabasi AL. Understanding individual human mobility patterns. Nature. 2008;453(7196):779–782.

[4] Android Developers: Manifest.permission;. http://bit.ly/1og9dUe.

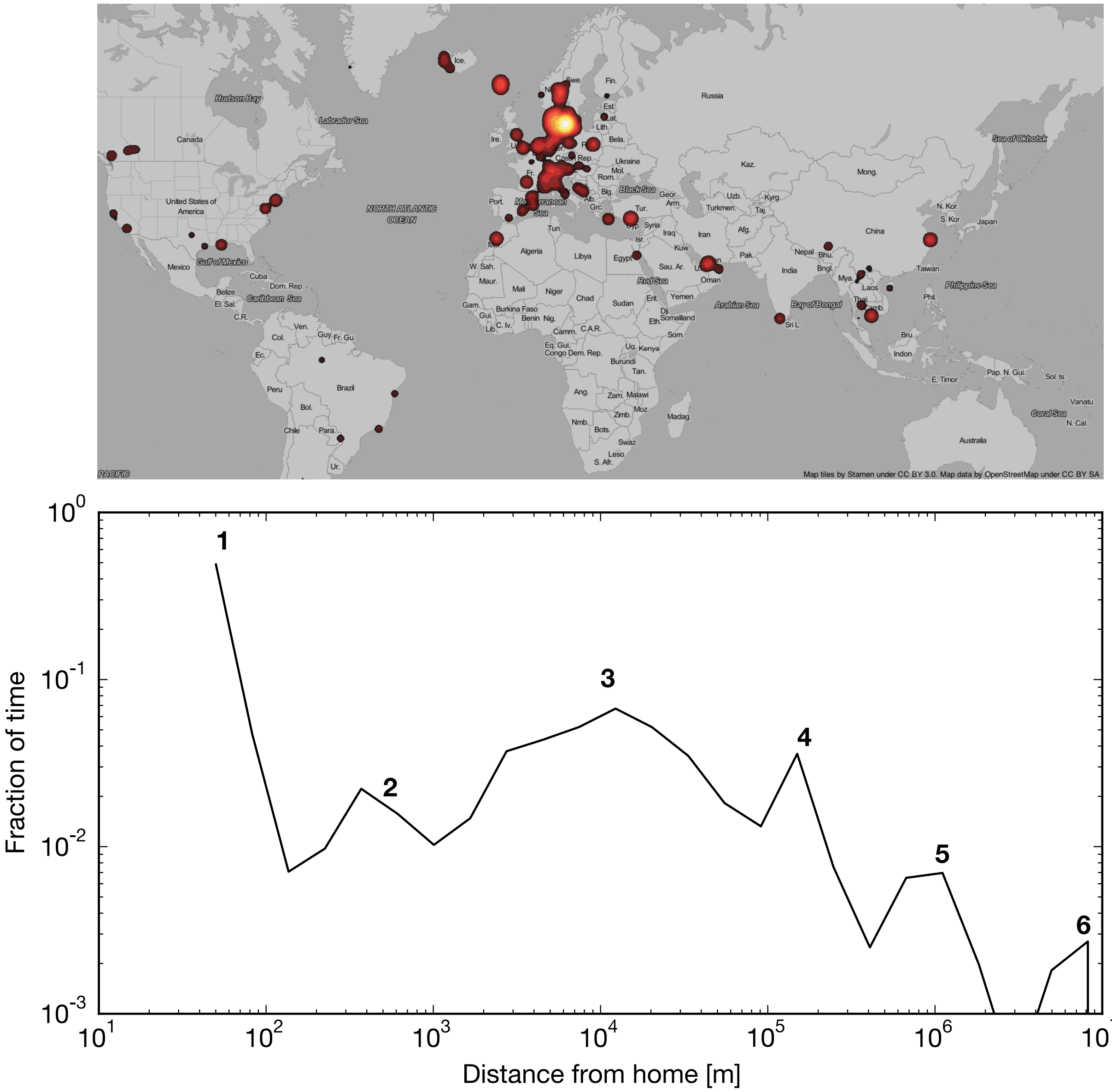

Fig B: The article focuses on a population of students at a single university, but they are not constrained to the campus only. Our data captures human mobility at different scales: the participants spend most of their time at home (1), but they travel around the neighborhood (2), the city (3), to different cities in Denmark (4), different cities in Europe (5), and finally, other continents (6).

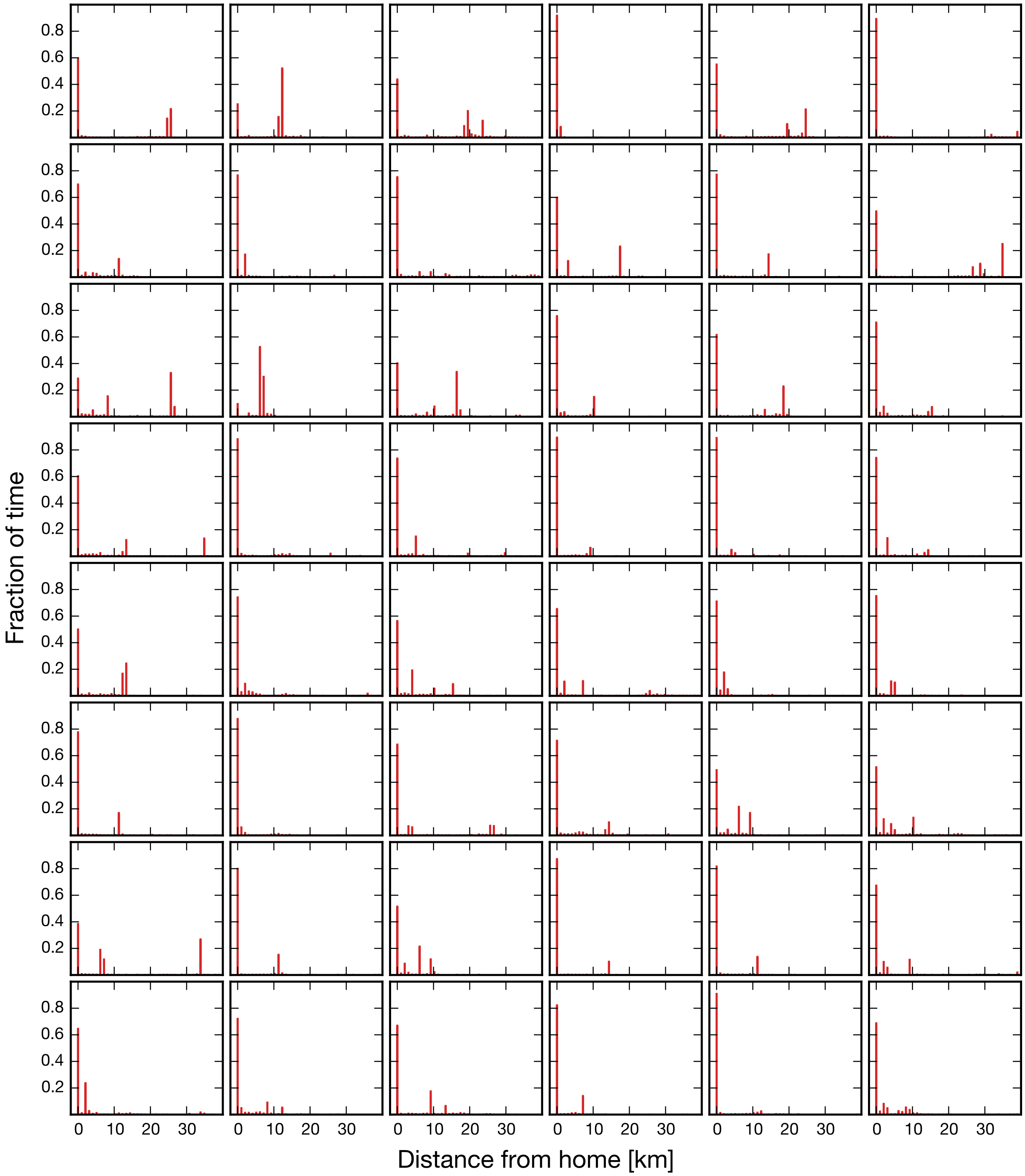

Fig C: Distribution of time spent at different distances from the inferred home location, presented for randomly selected 48 participants. In most cases, we see the home location as the most prevalent, and probably a "work" location as the next peak in the distribution.

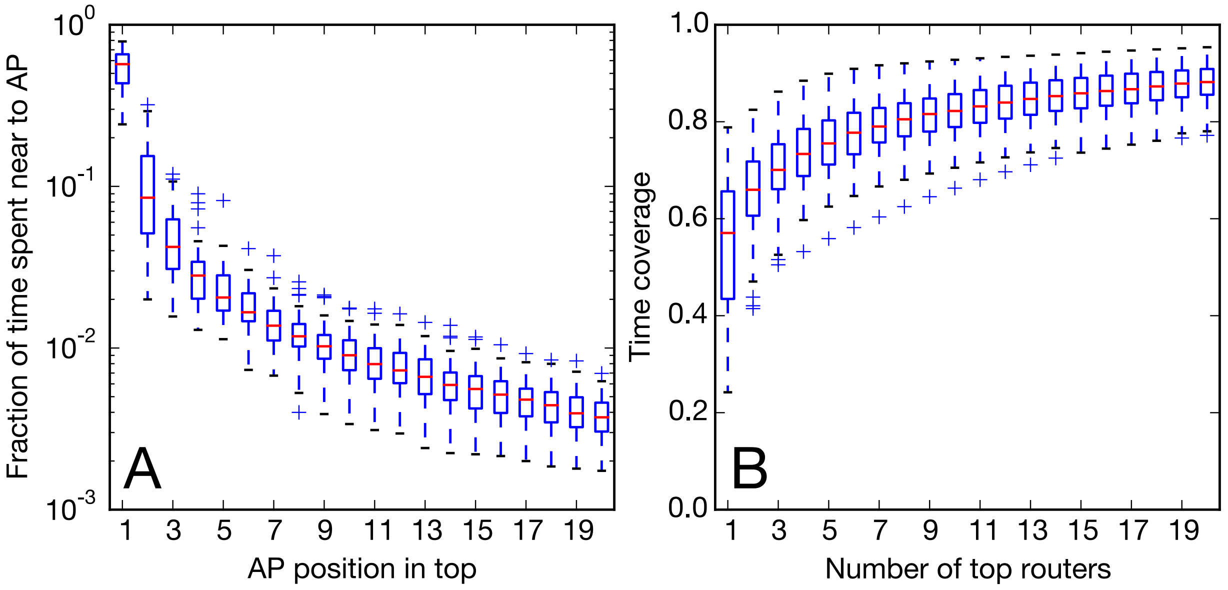

Fig D: A more detailed view of time coverage provided by top routers found through the greedy algorithm. A: there is a clear main location for a majority of participants, we therefore assume this to be the home location. B: even though 20 routers are needed on average to capture 90% of mobility, there are participants for whom just four routers suffice.
